# Device‐related differences and interchangeability of forced expiratory volume in 1 s measurements: A comparison of three spirometers

**DOI:** 10.1111/cpf.70105

**Published:** 2026-11-05

**Authors:** Leon L. Csonka, Antti Tikkakoski, Jussi Karjalainen, Lauri Lehtimäki

**Affiliations:** ^1^ Faculty of Medicine and Health Technology Tampere University Tampere Finland; ^2^ Department of Clinical Physiology and Nuclear Medicine Tampere University Hospital Tampere Finland; ^3^ Allergy Centre Tampere University Hospital Tampere Finland

**Keywords:** asthma, device comparison, laboratory spirometer, lung function, microspirometer, spirometry

## Abstract

**Background:**

Forced expiratory volume in 1 s (FEV_1_) obtained using different types of laboratory spirometers and handheld microspirometers is often used interchangeably in research, despite substantial differences between devices. The aim of this study was to assess device‐related differences and the interchangeability of FEV_1_ measurements among three commonly used spirometers.

**Methods:**

We recruited 20 healthy adults experienced in performing spirometry. Each participant performed three measurements with each of the three spirometers: the handheld microspirometers Medikro Duo and MIR Spirobank, and the laboratory spirometer Vyntus Pneumo.

**Results:**

In the linear mixed‐effects model, there was a significant main effect of device on FEV_1_ (F(2,38) = 25.25, *p* < 0.001), indicating that FEV_1_ values differed between devices. Spirobank yielded the highest FEV_1_ values, whereas the Medikro Duo yielded the lowest. The mean difference in FEV_1_ between Medikro Duo and Spirobank was −0.25 L (−0.34 to −0.16, *p* < 0.001), and between Medikro Duo and Vyntus Pneumo −0.16 L (−0.25 to −0.07, *p* < 0.001). The comparison between Spirobank and Vyntus Pneumo showed a smaller difference of 0.09 L (0.00 to 0.18), which narrowly missed statistical significance (*p* = 0.050). Bland–Altman plots showed wide limits of agreement between spirometers, suggesting poor interchangeability.

**Conclusions:**

Systematic bias and varying agreement between the laboratory spirometer and the handheld microspirometers indicate that FEV_1_ measurements obtained with different devices should not be assumed to be fully interchangeable. This does not imply that handheld microspirometry is inferior, but rather that device‐specific characteristics must be considered when interpreting and comparing FEV_1_ values.

## 1 INTRODUCTION

Asthma is a chronic respiratory disease characterized by variable expiratory airflow limitation. Objective assessment of lung function is central to its diagnosis, most commonly using spirometry. Spirometry provides several lung function parameters, of which forced expiratory volume in 1 s (FEV_1_) is the most important. Changes in FEV_1_ are used in clinical practice for diagnostic purposes, including bronchodilator response testing and bronchial challenge testing (GINA, 2025).

Recently, handheld microspirometers have become increasingly available and affordable, enabling spirometry to be performed at home. This may allow diagnostic home spirometry monitoring in the future, in which FEV_1_‐based diurnal variation and bronchodilator response may become important parameters (Myers et al., 2025). In both clinical practice and research, FEV_1_ values obtained using different devices are often compared or used interchangeably. However, spirometers differ in several respects, including sensor technology, sampling frequency, signal processing, flow resistance, and calibration, which may lead to systematic differences in measured FEV_1_ (Graham et al., 2019).

It is important to determine whether FEV_1_ values obtained from different types of spirometers differ and whether they can be used interchangeably. Of particular interest is how well handheld microspirometers agree with laboratory spirometry and how well different microspirometers agree with each other. Although some previous studies have compared FEV_1_ measurements obtained using different devices (Degryse et al., 2012; Liistro et al., 2006; Milanzi et al., 2019), not all devices currently in clinical use have been adequately evaluated. The aim of this study was therefore to assess device‐related differences and the interchangeability of FEV_1_ measurements obtained with a laboratory spirometer and two handheld microspirometers.

## 2 METHODS

We recruited 20 healthy volunteers experienced in performing spirometry from the staff of Tampere University Hospital. Spirometry was performed using three different devices: the handheld microspirometers Medikro Duo M920 (Medikro Oy) and MIR Spirobank II Smart (Medical International Research S.p.A.), and the laboratory spirometer Vyntus Pneumo (Jaeger Medical GmbH). Technical characteristics of the devices are presented in Table 1. Medikro Duo and Spirobank did not require user calibration, whereas Vyntus Pneumo was calibrated according to the manufacturer's recommendations.

**Table 1:** Technical properties of the three spirometers studied: Medikro Duo M920, MIR Spirobank II Smart, and Vyntus Pneumo.

Characteristic	Medikro Duo	MIR Spirobank	Vyntus Pneumo
Device type	Handheld microspirometer	Handheld microspirometer	Laboratory spirometer
Sensor technology	Disposable pneumotachograph	Bidirectional digital turbine	Heated pneumotachograph
Flow range	±14 L/s	±16 L/s	±20 L/s
Volume range	14 L	10 L	20 L
Flow accuracy	±5.5% or 0.25 L/s	±5% or 0.20 L/s	0–18 L/s: ±2%; 18–20 L/s: ±5%
Volume accuracy	±3.5% or 0.06 L	±2.5% or 0.05 L	±3% or 0.05 L
Flow resistance	0.8 cmH_2_O/(L/s) at 14 L/s	<0.5 cmH_2_O/(L/s) at 12 L/s	<0.5 cmH_2_O/(L/s) at 10 L/s
Calibration requirement	Not required	Not required	Syringe calibration
BTPS correction	Automatic	Automatic	Automatic

All measurements were performed under supervision. Each participant performed measurements with the three devices in randomized order. Participants performed at least three expiratory manoeuvres with each device, with additional manoeuvres performed when necessary to achieve repeatability. Participants were allowed to rest between devices if needed, and all measurements were completed within a maximum of 2 h. Technical acceptability and repeatability of FEV_1_ measurements were assessed according to European Respiratory Society criteria (Graham et al., 2019). All manoeuvres retained for analysis fulfilled the acceptability criteria, and all participant‐device measurement sets fulfilled the repeatability criteria. To reduce manoeuvre‐to‐manoeuvre variability and provide a stable estimate of FEV_1_ for device comparison, the mean of three measurements was used as the representative FEV_1_ value for each participant and device.

Data analysis was performed using IBM SPSS Statistics for Windows, Version 29 (IBM Corp.). The effects of the measurement device on FEV_1_ were analyzed using a linear mixed‐effects model with device as fixed effect and subject as a random effect. Model parameters were estimated using restricted maximum likelihood. Bland–Altman plots were created to further assess the interchangeability of measurements between all device pairs. For the linear mixed‐effects model, unadjusted *p*‐values were used, and *p* < 0.05 was considered statistically significant. For post hoc pairwise comparisons, Bonferroni‐adjusted *p*‐values were used, with adjusted *p*‐values < 0.05 considered statistically significant.

## 3 RESULTS

Table 2 presents the estimated marginal means of FEV_1_ by measurement device derived from the linear mixed‐effects model with device as fixed effect and subject as random effect. There was a significant main effect of device on FEV_1_ (F(2,38) = 25.25, *p* < 0.001), indicating that FEV_1_ values differed between devices. Spirobank yielded the highest FEV_1_ values, whereas Medikro Duo yielded the lowest.

**Table 2:** Estimated marginal means (95% confidence intervals) of forced expiratory volume in 1 s (FEV_1_) by measurement device derived from the linear mixed‐effects model with device as fixed effect and subject as random effect.

Device	Mean FEV_1_, L (95% CI)
Medikro Duo	3.39 (3.09 to 3.69)
Spirobank	3.64 (3.34 to 3.94)
Vyntus Pneumo	3.55 (3.25 to 3.85)

We also assessed pairwise differences in FEV_1_ between spirometers. The mean difference in FEV_1_ (95% CI) between Medikro Duo and Spirobank was −0.25 L (−0.34 to −0.16, *p* < 0.001), and between Medikro Duo and Vyntus Pneumo −0.16 L (−0.25 to −0.07, *p* < 0.001). The comparison between Spirobank and Vyntus Pneumo showed a smaller difference of 0.09 L (0.00 to 0.18), which narrowly missed statistical significance (*p* = 0.050). Bonferroni correction was applied to adjust *p*‐values for multiple comparisons.

Figure 1 presents Bland–Altman plots of FEV_1_ measurements illustrating the agreement between the three spirometers. The 95% limits of agreement (LoA) were relatively wide in all comparisons, but there were differences between pairs. The Medikro Duo and Spirobank pair showed the widest LoA (−0.62 to 0.11 L), suggesting particularly poor interchangeability (Figure 1A). Although the mean bias deviated slightly more from zero between Medikro Duo and Vyntus Pneumo (−0.16 L) than between Spirobank and Vyntus Pneumo (0.09 L), the LoA between Spirobank and Vyntus Pneumo were wider (−0.25 to 0.43 L; Figure 1C) than for Medikro Duo and Vyntus Pneumo (−0.38 to 0.06 L; Figure 1B). No clear proportional bias was observed in any of the pairs.

**Figure 1:**
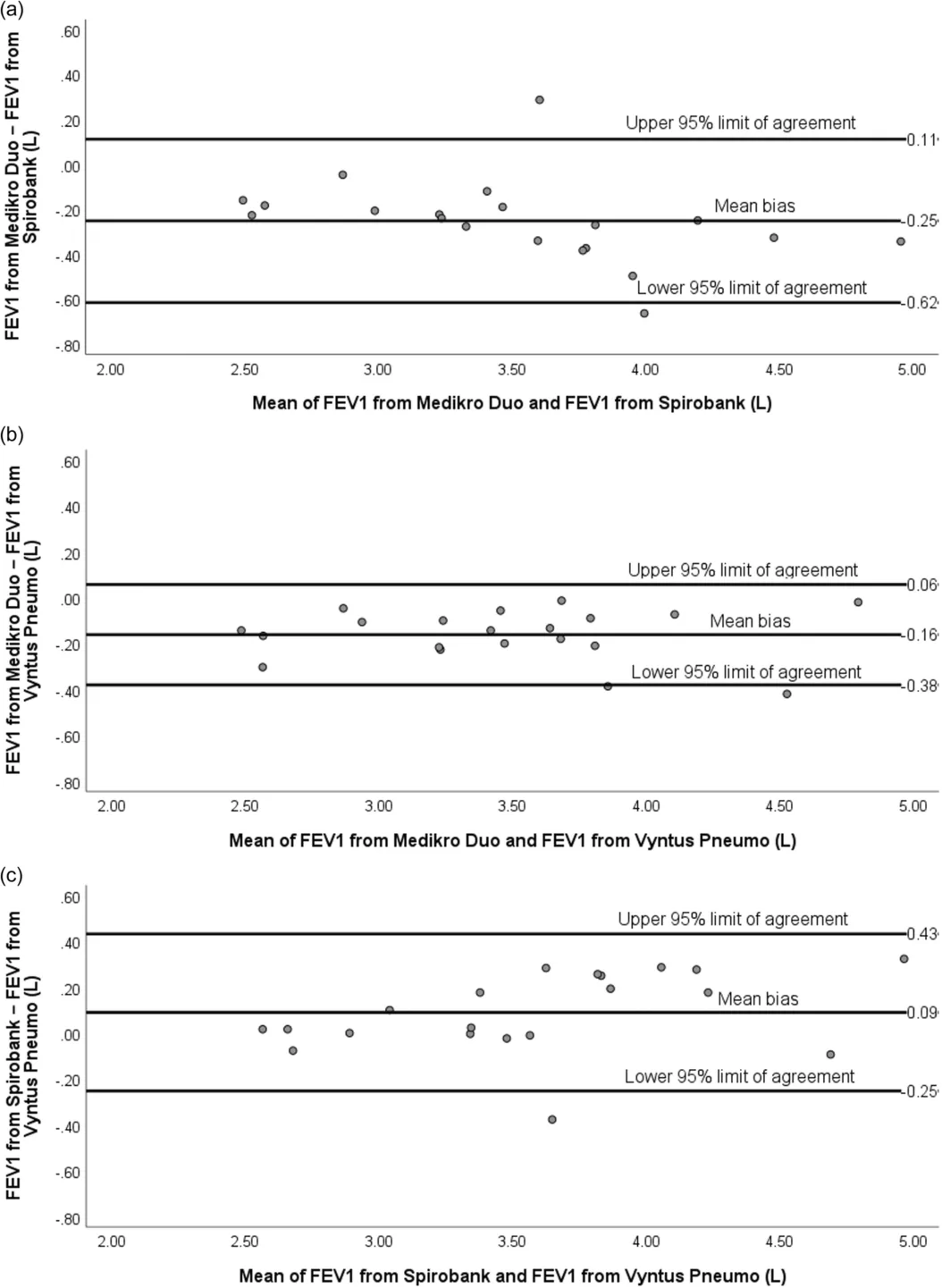
Bland–Altman plots of forced expiratory volume in 1 s (FEV_1_) measurements illustrating the interchangeability of three spirometer pairs: Medikro Duo and Spirobank (a), Medikro Duo and Vyntus Pneumo (b), and Spirobank and Vyntus Pneumo (c).

## 4 DISCUSSION

In this study, we found significant device‐related differences in FEV_1_ measurements. Among the handheld microspirometers, Spirobank showed a smaller mean bias relative to the laboratory spirometer Vyntus Pneumo, whereas Medikro Duo demonstrated narrower limits of agreement. However, the limits of agreement were relatively wide for all device pairs, and the greatest disagreement, both in terms of mean bias and limits of agreement, was observed between Medikro Duo and Spirobank. Therefore, although the two handheld devices slightly differ in the pattern of disagreement with the laboratory spirometer, neither can be considered fully interchangeable with it or with each other.

Technical differences between the spirometers studied may help explain the variation observed in FEV_1_ measurements. Vyntus Pneumo and Medikro Duo employ different forms of pneumotachograph‐based flow measurement (Jaeger Medical GmbH, n.d.; Medikro Oy, n.d.), while Spirobank uses a bidirectional digital turbine flow sensor (Medical International Research, n.d.). These differing measurement principles may contribute to systematic differences between devices, as pneumotachographs are particularly sensitive to calibration and environmental factors, such as temperature and moisture (Miller & Sigsgaard, 1994), whereas turbine‐based devices are affected by mechanical inertia (Yeh et al., 1987). In addition, differences in signal processing algorithms and sampling rates may further influence measurement outcomes, although detailed information regarding these characteristics is generally proprietary to manufacturers.

The findings of this study are particularly important for research settings in which FEV_1_ values obtained from different devices are often used interchangeably. With growing recognition that peak expiratory flow (PEF) is a physiologically inferior parameter for detecting bronchoconstriction and bronchodilation, and with the increasing availability and affordability of handheld microspirometers, interest in home FEV_1_ monitoring has risen as an alternative to home PEF monitoring (Csonka et al., 2024, 2025). Studies are currently needed and underway to assess the diagnostic accuracy of monitoring diurnal FEV_1_ variation and bronchodilator response at home (Myers et al., 2025). In light of our results, direct comparison or pooling of home and laboratory FEV_1_ values in such studies may not be appropriate.

If absolute home‐based FEV_1_ values are compared directly with clinic‐based values, mean bias may be particularly important, which would favour Spirobank because of its smaller systematic difference relative to Vyntus Pneumo. In contrast, the narrower limits of agreement between Medikro Duo and Vyntus Pneumo indicate less variability in the differences between these devices at the individual level, despite a larger systematic bias. This suggests that relative changes in FEV_1_, such as diurnal variation or bronchodilator response, may be reflected more consistently by Medikro Duo and laboratory spirometry than by Spirobank and laboratory spirometry. However, this interpretation should be made with caution, as the present analysis was not specifically designed to assess agreement in longitudinal changes.

We were unable to find relevant studies for the specific Medikro Duo M920 model used in our study. An earlier Medikro microspirometer, SpiroStar, was evaluated together with Spirobank and eight other microspirometers in a multicenter study by Liistro et al., who found that the mean bias in FEV_1_ for both microspirometers compared with laboratory spirometry was not statistically significant (Liistro et al., 2006). Spirobank was also assessed in a study by Degryse et al. and was found to slightly underestimate FEV_1_ compared with the MasterScope laboratory spirometer (Degryse et al., 2012). The results of these studies regarding Spirobank somewhat contradict our findings, as we observed that Spirobank tended to yield higher FEV_1_ values than laboratory spirometry, although statistical significance was narrowly missed (*p* = 0.050). These differences may be explained by the use of different laboratory spirometers and by other methodological differences between the studies (Degryse et al., 2012; Liistro et al., 2006).

In the broader literature, handheld microspirometers often show at least a small systematic bias relative to laboratory spirometry, most commonly in the direction of lower FEV_1_ values (Degryse et al., 2012; Milanzi et al., 2019; Puri et al., 2011). In this context, it is noteworthy that in our study, Medikro Duo conformed to this pattern, whereas Spirobank showed the opposite behaviour. Oppenheimer et al. compared FEV_1_ measurements obtained by unsupervised home spirometry (AM3 device) with laboratory spirometry (MasterScope spirometer) using Bland–Altman analysis, among other methods, and found poor interchangeability, with substantially wider limits of agreement than those observed in our study (Oppenheimer et al., 2023). However, the unsupervised nature of home spirometry makes it difficult to disentangle the effects of measurement technique from true device‐related differences.

The strengths of this study include highly reliable measurements achieved through supervised testing, assessment of technical quality, and the inclusion of participants experienced in spirometry. In addition, the study evaluated understudied yet commonly used spirometers that meet ERS requirements (Graham et al., 2019). By using a linear mixed‐effects model, we accounted for subject‐level variability and the non‐independence of repeated measurements, and Bland–Altman analysis allowed a more detailed assessment of interchangeability. However, the sample size was modest, which means that the limits of agreement, in particular, should be interpreted with caution. In addition, generalizability may be limited by the inclusion of only adults, all of whom were healthy, experienced in spirometry, and recruited from a single centre. Nevertheless, these same factors also represent a strength in the context of the present study, as a healthy, experienced, and relatively homogeneous population reduces confounding related to technique and allows clearer isolation of device‐related effects. Only one laboratory spirometer and two handheld microspirometers were assessed, and therefore, the findings cannot be directly extrapolated to other devices.

In conclusion, the presence of systematic bias and varying agreement in FEV_1_ between the laboratory spirometer and the two handheld microspirometers studied indicates that FEV_1_ measurements obtained with different devices should not be assumed to be fully interchangeable. This should not be taken to suggest that handheld microspirometers are inferior to laboratory spirometers, but rather that device‐specific characteristics must be considered when interpreting and directly comparing FEV_1_ values.

## AUTHOR CONTRIBUTIONS

**Leon L. Csonka**: Conceptualization; data curation; investigation; formal analysis; methodology, visualization; writing—original draft; writing—review and editing. **Antti Tikkakoski**: Conceptualization; data curation; investigation; resources; writing—review and editing. **Jussi Karjalainen**: Conceptualization; writing—review and editing. **Lauri Lehtimäki**: Conceptualization; writing—review & editing; resources; project administration; supervision; funding acquisition.

## CONFLICT OF INTEREST STATEMENT

L.L.C. reports no conflicts of interest. A.T. has received personal fees for lectures from Boehringer Ingelheim, Chiesi, GSK and Orion Pharma. J.K. has received personal fees for advisory board meetings and lectures from Astra Zeneca, Boehringer Ingelheim, Chiesi, GSK, Orion Pharma and Sanofi. L.L. has received personal fees for advisory board meetings and lectures from ALK, Astellas Pharma, Astra Zeneca, Berlin Chemie, Boehringer Ingelheim, Chiesi, GSK, Orion Pharma and Sanofi.

## Data Availability

The data that support the findings of this study are available on reasonable request from the corresponding author. The data are not publicly available due to privacy or ethical restrictions.
